# Experimental and Numerical Determination of Strength Characteristics Related to Paraglider Wing with Fourier Transform Infrared Spectroscopy of Applied Materials

**DOI:** 10.3390/ma15207291

**Published:** 2022-10-18

**Authors:** Paulina Maślanka, Andrii Aleksieiev, Ryszard Korycki, Halina Szafrańska, Anna Dąbrowska

**Affiliations:** 1 Interdisciplinary Doctoral School, Lodz University of Technology, Zeromskiego 116, 90-924 Lodz, Poland; 2 Department of Mechanical Engineering, Informatics and Chemistry of Polymer Materials, Lodz University of Technology, Stefanowskiego 12/16, 90-924 Lodz, Poland; 3 Department of Physicochemistry and Materials Technology, Faculty of Chemical Engineering and Commodity Science, Kazimierz Pulaski University of Technology and Humanities in Radom, Chrobrego Str. 27, 26-600 Radom, Poland; 4 Department of Personal Protective Equipment, Central Institute for Labour Protection—National Research Institute (CIOP-PIB), Wierzbowa Str. 48, 90-133 Lodz, Poland

**Keywords:** Fourier transform infrared spectroscopy, aerodynamic characteristics, strength characteristics, air permeability, numerical modeling

## Abstract

The aim of paper is to determine experimentally and numerically the strength characteristics related to the paraglider wing with Fourier transform infrared spectroscopy of applied materials. The applied method consists in theoretical modeling supplemented by the tests of material parameters. First, the set of 10 lightweight fabrics was selected for the tests; the samples are representative for these structures. The materials were tested using the spectroscopy to determine the FTIR spectra. The samples differ in the content of certain characteristic groups. Air permeability change of the materials was determined for the different pressure drops. The air permeability of almost all the analyzed samples was close to zero with the exception of only one material. The tensile strength and elongation at the break of samples were determined on the testing machine. The paraglider samples were characterized by slightly decreased mechanical properties compared to the parachute fabrics. The material characteristics determined during the tests are the input data for the theoretical analysis. The numerical model of the paraglider wing is based on a 3D geometry from previous research, but the stress, strain, and deformation were determined using the ANSYS Structural program and the finite elements method. To determine the strength correctly, we introduce two basic values: the absolute maximal and the representative values that are the biggest repetitive values of stress, strain, and deformation. The stress value was determined by the main factors: (i) the thinner the material, the bigger the stresses that were accumulated; (ii) the stronger the material, the bigger the stresses that were accumulated. The results are similar for all materials and differ mainly by the values. The biggest stresses were observed inside the material contacting the ribs, whereas the biggest deformation and strain were in the regions between ribs, and the smallest were in the contact areas with the fixed supports. Their highest intensity was observed on the leading edge of the paraglider. We conclude that the obtained stresses were far from the breaking level for the wing.

## 1. Introduction

The analysis of state variables related to the lightweight paraglider wing in variable environmental conditions is relatively rare in the available scientific literature. The problem is difficult in respect to the complex structural geometry and variable flight conditions. The authors analyze the selected problems of paraglider aerodynamics and the obtained results focus on the particular solutions of dynamic problems; the references to the specific are compiled below.

The paraglider was first numerically analyzed in a sense of computational fluid dynamics by Zhu, Cao [1,2] in 2012. Then, the problem was consequently developed by other authors [3]. The research considered mainly geometric parameters, deflation of the wing, and pressure gradient along a paraglider wing.

Following this, authors started examining the purpose of quantifying the amount of aerodynamic drag related to the flexible nature of a paraglider wing. The drag of a pilot in a harness was estimated by means of wind tunnel testing, and a computational fluid dynamics solver was used to estimate smooth wing lift and drag characteristics [4].

An instrumentation system for in situ measurement of the pressure differential at the upper and lower surfaces of dynamically inflatable wings were designed and tested by Benedetti, Gurgel Veras [5]. The experimental data confirms the occurrence of a bottom leading-edge recirculation bubble, linked to the low Reynolds regime and the presence of an air intake.

Double-membrane gliding parachutes were analyzed by Ovchinnikov, Petrov, and Ganiev [6] in respect to the wide variety of application, including the solution of cargo transportation problems.

An inflatable cell model of paragrlider’s wing was investigated by Md. Nizam Uddin, Mohammad Mashud in wind tunnel experiments, which are designed to represent the dynamic behaviors of each cell comprising the canopy [7].

The article presented is a continuation of these previous works [8,9]. In paper [8] we defined two PA-fabrics covering a paraglider wing, with the comparative object being the permeable clothing material. The material parameters of textile materials are applied in numerical simulations of aerodynamic characteristics. The space model of the paraglider wing and the plane model of its cross-section in the symmetry plane are analyzed. The visualization of the stream filaments shows that it is advisable to cover the wing with the impermeable fabric.

Maslanka and Korycki [9] analyzed the sensitivity of aerodynamic characteristics to the properties of the material used for the paraglider wing. The covering material yields adequate pressure distribution between the external and internal parts of the wing. The finite volume mesh is defined using the ANSYS Meshing program. Numerical analysis uses different covering materials, ranging from the air-impermeable covering to the covering subjected to hydrolytic–mechanical degradation. Optimization of properties of the covering material improves the lift force and the aerodynamic characteristics of the wing.

Generally, the shape of the paraglider and material properties of the covering materials should be optimized [10,11]. The main difficulty is the proper formulation of optimization that is functional. The parameters that allow us to compare the materials covering the paraglider wing are the pressure (determination of lift force and stability problems), as well as the stresses in the materials created during the flight. The other problem is the connection of wing components by seams [12,13]. The alternative is the formulation of the micro-scale model [14] or the numerical optimization using the set of subsequent solutions [15].

The main idea of the paper presented is to analyze the selected lightweight paraglider and parachute fabrics made of polyamide 6.6 and coated with polyurethane resins or silicone, to determine their air permeability and strength characteristics. The laboratory testing on scaled models is difficult because of problems in the elastic similitude of compared structures. On the other hand, tests of full-scale models in a large facility with a large full-scale test section are expensive. The state variables are now the pressure in case of flight characteristics and the strength parameters during the analysis of material effort; that is, the stress, strain, and displacement.

When analyzing the obtained results of Fourier transform infrared spectroscopy, it can be concluded that samples differ in the content of certain characteristic groups. The determination of the functional groups present in the samples was crucial for the selection of four samples, the parameters of which were later entered into the simulation. Moreover, the analysis of the peaks obtained during the FTIR spectroscopy analysis could give an overview of the expected strength properties of the considered samples.

Air permeability change of the analyzed samples was determined according to the standards EN ISO 9237:1995 [16] and PIA-C-44378 [17]. The pressure drops equal to: 100 Pa, 125 Pa, 1500 Pa, 2000 Pa, and 2500 Pa were used to compare the air permeability change of the samples. According to the analyzed overloads during the flight, the materials are subjected to the maximal pressure drop equal to 1036 Pa. Therefore, the test results of air permeability for a pressure drop equal to 1500 Pa were chosen to be the most proper for the further analysis. Next, the tensile properties (represented by the tensile strength and elongation at break [18]) were determined using the testing machine. The tests were performed in the normal climate conditions; that is T = 20 °C; p = 1013.25 hPa; and RH = 65%.

Finally, the anisotropic characteristics of the woven materials that were achieved and analyzed in the course of physical testing were the input data in the ANSYS Structural program. The geometrical model is approximated by the dimensionless coordinates of crucial points inside the wing and smoothed by spline curves. The finite element mesh is defined using the ANSYS Meshing program. The obtained distribution of stress, strain, and displacement are comparable but the values are completely different.

Numerical tests and simulations are essential to determine the distributions of pressure and stresses inside the paraglider wing. These parameters affect the flight stability and consequently the user’s safety

The novelty elements can be formulated as follows: (i) The analysis of a lightweight paraglider wing is still innovative, particularly in respect to different state variables, i.e., the pressure in flight characteristics problems; such as the stress, strain, and displacement during investigation of the material properties. (ii) The selected lightweight paraglider and parachute fabrics made of polyamide 6.6 coated with polyurethane resins or silicone are analyzed to determine their air permeability and strength characteristics with the Fourier transform infrared spectroscopy. The infrared spectroscopy is infrequently investigated in case of coated textile materials, particularly the lightweight textiles specific for a paraglider. (iii) The air permeability is difficult to determine because almost all the analyzed samples are impermeable. Thus, the materials should be tested for different pressure drops to determine correctly the relationship between the permeability and the pressure. (iv) The article can provide reference for further studies on optimal material parameters and their coincidence with the flight parameters of a paraglider wing.

## 2. Materials

The analyzed materials are lightweight paraglider fabrics (Table 1, no. 1–8) and parachute fabrics (Table 1, no. 9–10) made of polyamide 6.6, which are usually coated with polyurethane resins or silicone. The fabrics were received thanks to manufacturers from RPA and Poland. However, due to their confidential character, the authors were asked not to give names of the manufacturers, nor the trade names of the samples.

The exact characteristic functional groups were studied in Section 3.1.1 and Section 4.1.

They are all manufactured using a standard ripstop weave (creating visible squares). Only one of the analyzed fabrics (no. 9) has a special hexagonal ripstop weave, which causes increased dimensional stability when stretched in different directions.

Obtained mass of the fabrics are of a range of 26–42 g/m^2^ and their thickness is of a range of 0.05–0.09 mm (Table 1). All the samples are of air-permeability tending toward zero, which was studied and described in the following Section 3.1.2, Section 3.1.3 and Section 4.2.

## 3. Methods

### 3.1. Experiments

#### 3.1.1. Fourier Transform Infrared Spectroscopy (FTIR)

FTIR spectra were recorded by a Nicolet 8700 spectrophotometer (Thermo Fischer Scientific Instruments, Waltham, MA, USA), with a diamond adapter equipped (Smart Orbit ATR sampling accessory), and for better accuracy of the analysis of modified samples, 128 scans were taken in the range of 3800–800 cm^−1^.

#### 3.1.2. Air Permeability

Air permeability change of the analyzed samples was determined by the FX 3300 digital device and based on the EN ISO 9237:1995 standard [16]. The standard pressure drop between the two sides of a textile fabric is 100 Pa. However, according to the literature and product characteristics given by the paraglider fabric manufacturers, the air permeability for this kind of material is usually determined with a pressure drop equal to 2000 Pa. On the other hand, according to the PIA-C-44378 standard (parachute cloth specification) [17], the pressure drop should be equal to 125 Pa. Therefore, the pressure drops are equal to 100 Pa, 125 Pa, 1500 Pa, 2000 Pa, and 2500 Pa were used to compare the air permeability change of the analyzed samples. According to the standard, the surface area of 20 cm^2^ was applied. The results are defined in mm/s. The tests were performed in the normal climate conditions, i.e., T = 20 °C; p = 1013.25 hPa; and RH = 65%.

#### 3.1.3. Tensile Properties

An Instron Series 5560 device equipped with a computer program of the TX Series was used to analyze the tensile strength and elongation at break. According to the ISO 13934-1:2013 [18], the width of the sample was equal to 50 mm, the distance between clamps was 200 mm, and the speed rate of the clamps equal to 100 mm/min was applied. The tests were performed in the warp and the weft direction in the normal climate conditions, i.e., T = 20 °C; p = 1013.25 hPa; and RH = 65%.

### 3.2. Simulations

Stress, strain, and deformation distributions over a 3D model of a paraglider were determined using the ANSYS Structural program and the finite elements method (FEM).

The solution was based on the Newton–Raphson method, which is used for calculation of the force displacement curve for a nonlinear system. It is done by assuming a zero displacement system and then implementing iterations of forces and displacements until there is a convergence (which is assumed according to convergence criteria).

Based on the literature [19], when simplified, the Newton–Raphson method is described by the following equations, where (1) is the displacement increment and (2) is the residual force:

(1) $$\Delta u^{n}=\left(F_{ext}-F_{int}^{n}\right)/K^{n}$$

(2) $$F_{res}^{n}=F_{ext}-F_{int}^{n}$$

where:

*N*—iteration

*F_ext_*—applied force

*F_int_*—computed internal force

*F_res_*—residual force

Δ*u*—displacement increment

*K*—stiffness matrix

The convergence is achieved when $F_{res}^{n}\leq convergence\;criteria$.

The model is based on a 3D geometry from previous research [9]. However, it differs because of the type of calculation (the previous was a *Computational Fluid Dynamics*, further CFD type).

The ANSYS Structural program uses input data for pre-processing. The input data can be, for example, material properties, geometry, supports, or forces acting on a considered material. Some of them (mainly the material parameters) are provided thanks to the previously described testing methods, i.e., density, thickness, Young’s modulus, and tensile strength. The air permeability parameter is not directly implemented into the ANSYS Structural; however, based on this property, definition of pressure distribution over the paraglider (which certainly is the input data for the considered case) is possible. Properties of materials obtained in the course of the experimental testing described below were a basis for the theoretical analysis and obtaining the distributions of stress, strain, and deformation considered in the following sections.

Tensile forces acting on the covering materials of a paraglider are created by the pressure difference between air inside a paraglider wing and around it. The pressure distribution over a paraglider covered with impermeable and permeable materials were determined using the ANSYS Fluent program during the previous analysis mentioned above [8,9] and the maximal overload that can be achieved.

## 4. Experimental Results

### 4.1. Fourier Transform Infrared Spectroscopy (FTIR)

The Fourier transform infrared spectroscopy is widely discussed in available literature in respect to different applications. The main problem is the external coating of the paraglider fabrics, which can influence the test methodology, as well as the strength properties of the material. The problem is discussed in [20,21,22,23,24,25] for different coatings and base materials. The infrared spectroscopy is infrequently investigated in case of coated textile materials [21].

**Figure 1:**
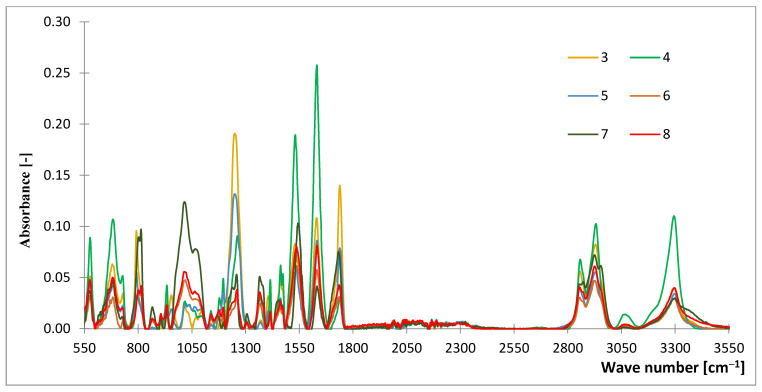
The FTIR spectra of the analyzed samples (3–8).

**Figure 2:**
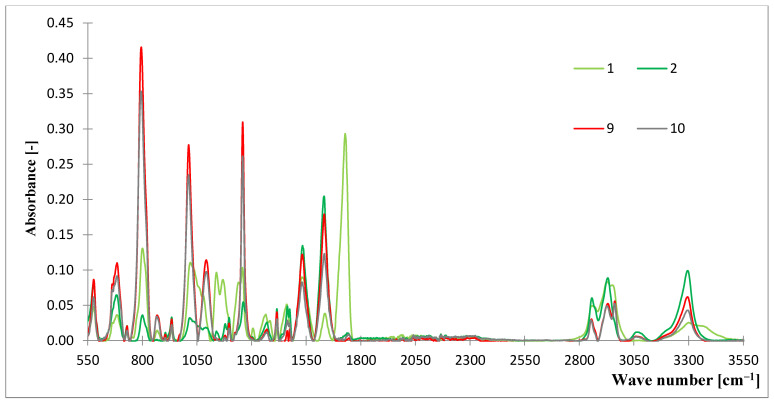
The FTIR spectra of the analyzed samples (no. 1–2, 9–10).

The FTIR spectra of the samples are presented in Figure 1 and Figure 2 and the absorption ranges corresponding to the characteristic bonds are presented in Table 2. When analyzing the obtained FTIR spectra, it can be concluded that samples differ in the content of certain characteristic groups [22].

The sample no. 1 is distinguished from other samples due to the presence of some characteristic peaks, which were not registered for any other tested material. A broad band at 1183–1153 cm^−1^ with a peak at 1169 cm^−1^ and 1153–1123 cm^−1^ with a peak of 1138 cm^−1^ are related to the presence of C–O stretching vibrations and to the axial stretching of the C–N–C bond, which is characteristic for polyamide 66 [23]. A recorded peak at 1735–1690 cm^−1^ corresponding to the C=O group, indicates its higher content in material compared to the remaining samples [21].

In the case of the samples 9 and 10, the maximum intensity of peaks, and as a result the highest content of assigned bonds, were recorded for the following groups: –CH_3_, tensile of Si–C in Si–CH_3_ at 796–789 cm^−1^, tensile of Si–O–Si at 1074–1005 cm^−1^ and CH_3_ strain in Si–CH_3_ at 1260–1250 cm^−1^. It should also be noticed that the vibrations characteristic for the CH, CH_2_, and CH_3_ groups occurred within the range of 3100–2852 cm^−1^, and the characteristic N–H group at 3300–3330 cm^−1^ [24,25].

Analysis of the peaks obtained during the FTIR spectroscopy test could give an overview of the expected strength parameters of the samples. Higher peaks often indicate functional groups that give the material greater mechanical strength.

In the case of sample numbers 9 and 10, these are the Si–C, –Si–CH3 groups; therefore, these samples are characterized by the higher strength parameters. Another example can be sample no. 5, which is characterized by the lowest number of O–C–O, C–O–C, C–OH, and C–H groups. Sample no. 1 is the only described by the largest recognizable peak near the group C=O and C–O.

Simultaneously, the present groups are caused by chemical bondings, which affect the strength of the considered materials. When single bonding is considered, it is too weak to have any influence. However, when a composite is considered, their overall impact can be significant to the strength characteristics.

According to the Table 2, the specific, hydrogen bonds (–H) were compiled. These covalent (polarized bonds) resulting at the end of the considered groups may interact with the elements of other compounds and groups (e.g., oxygen = O). Other, specific types of bonds are those created by O and N; they create multiple bonds. N creates coordination bonds, whereas Si creates polarized covalent bonds. The authors aimed to investigate the type and nature of the interactions resulting from functional groups; therefore, they did not describe detailed analysis, but rather focused on the improved properties of the modified samples of considered fabrics.

### 4.2. Air Permeability and Strength Characteristics

**Figure 3:**
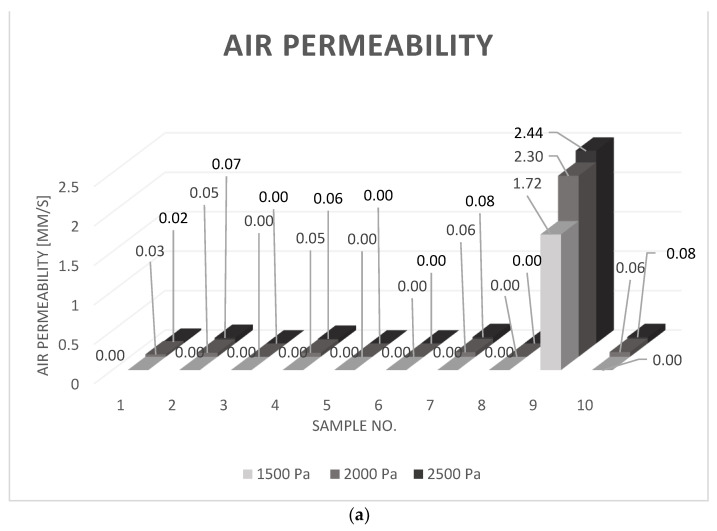

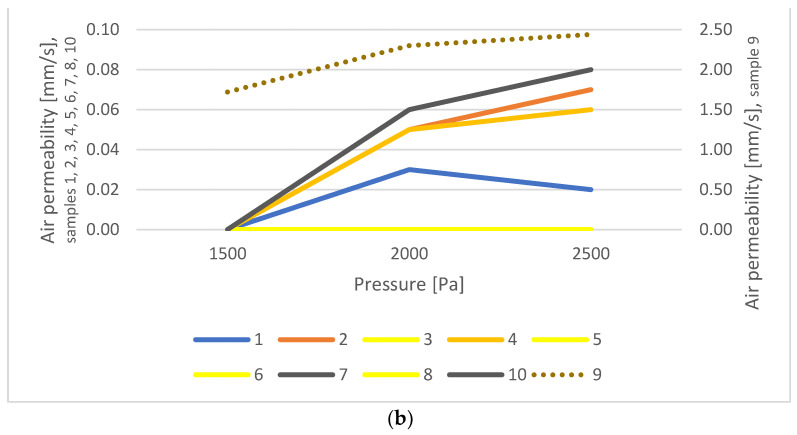
Obtained air permeability values, with pressure drops: 1500 Pa, 2000 Pa, 2500 Pa; (**a**)—3D bar chart; and (**b**)—2D linear graph with two air permeability ranges.

According to the results listed in Table 3 and Figure 3, the air permeability values of almost all the analyzed samples were close to zero. The only sample characterized by a higher air permeability value was sample number 9. All the other samples were completely impermeable when pressure drop was 100 Pa, 125 Pa, and 1500 Pa. For the increased pressure drops, samples no. 1, 2, 4, 7, and 10 reached values of the air permeability greater than 0 mm/s; however, the results are at the hundredth order of magnitude.

As mentioned above, sample no. 9 was characterized by the highest air permeability values, which were between 1.72 and 2.44 mm/s (when the pressure drop was 1500–2500 Pa). Regardless of the fact that it is an infinitely greater result than for the other samples, the air permeability of this sample is still very low. The results are equal to 0 mm/s when tested according to ISO 9237:1995 [16] and PIA-C-44378 [17].

**Figure 4:**
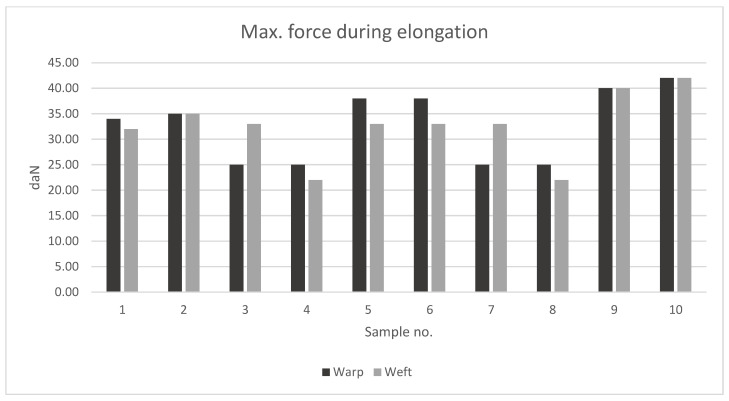
Obtained values of the breaking force during the tensile tests.

Based on the Table 3 and Figure 4, the maximal forces during elongation were the greatest for the samples no. 9 and 10 (parachute fabrics); their values were between 40 and 42 daN.

The paraglider samples were characterized by slightly decreased mechanical properties compared to the parachute samples. The highest values presented by samples no. 2, 5, and 6 (the breaking forces were around: 35 daN). The lowest mechanical strengths were presented by samples no. 4 and 8 (value of force around 25 daN and less). Increasing the value of breaking forces is associated with increasing mass of the samples.

Due to the biggest differences between characteristics of fabrics 4, 6, 9, and 10, they were chosen for further calculations in the ANSYS Structural program.

In order to exploit the above-described strength characteristics for the further computational calculations, it was necessary to determine Young’s modulus of the chosen materials. Young’s modulus is the most important indicator for this type of calculation; the others are density, thickness, tensile strength, Poisson’s ratio, and shear modulus. However, the available laboratory tests did not allow for determination of the two last parameters in a simple way. As, in fact, they do not influence the final results significantly, the Poisson’s ratio and Shear modulus were assumed based on the literature regarding other nylon fabrics with similar characteristics [26,27].

Young’s modulus is a mechanical property describing the stiffness of a considered material when a force is applied lengthwise. Its formula is the following:

(3) $$E=\frac{\sigma }{\varepsilon }$$

where:

*E*—Young’s modulus, pressure units

*σ*—Uniaxial stress, pressure units

*ε*—Strain, dimensionless

The stress (*σ*) is calculated based on the force and the area of the sample; the area can be calculated based on the width and the thickness of the considered sample.

The force and elongation values were compiled in the Table 3.

**Figure 5:**
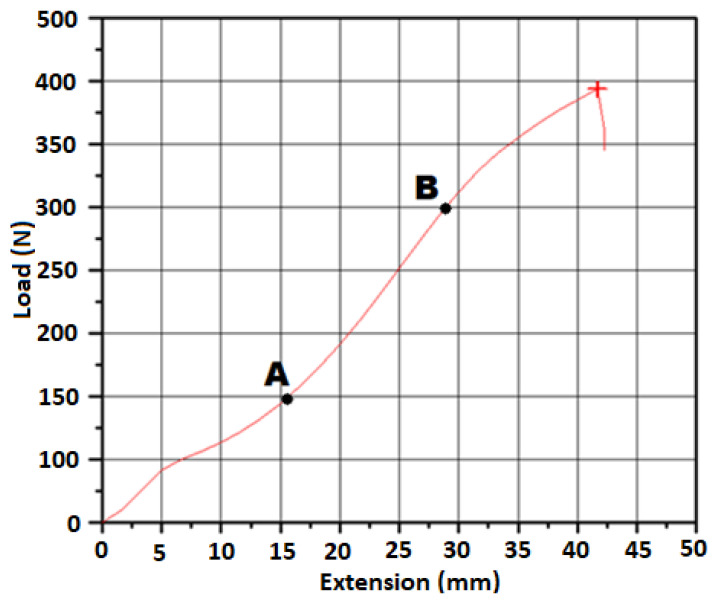
The exemplary graph for calculation of the Young’s modulus (sample 1, warp).

However, based on Figure 5, the tensile curve graph has no linear characteristic from the starting point of the test. It is caused by a characteristic structure of woven materials in which the yarns interlace each other [27]. The first section of the graph (before point A) describes straightening of the yarn before the actual tensile.

Simultaneously, the values of Young’s modulus obtained for forces greater than indicated after point B presented in the graph were not applicable to the considered case, which was proven by further calculations. For example, for material 6, the stress in point B was between 60 and 70 MPa (depending on considered direction, i.e., warp/weft), whereas maximum stress obtained during calculations was 18.37 MPa (Table 6 in the result section), which is a significantly lower value.

Therefore, the Young’s modulus was calculated for the A-B section, where the graph presents linear characteristics of the tensile test.

The Young’s modulus values of the all considered samples were calculated based on the above described scheme. The obtained Young’s modulus values were compiled in Table 4 below.

Based on Table 4, it can be observed that the Young’s modulus (E) values were comparable for both directions of one sample; however, for the considered cases, the bigger values of *E* were always in the warp direction. The biggest difference between the linear elasticity of warp and weft was noticed to be for the sample no. 6 (the linear elasticity value into the weft direction decreased around 8% compared to the warp direction). It is closely related to characteristic structure of woven materials, which gives them anisotropic properties. The elasticity modulus’ increase/decrease into different directions of a material can be caused by, e.g., difference in the number of threads per unit of length.

## 5. Simulations

### 5.1. Modeling of Paraglider Wing

**Figure 6:**
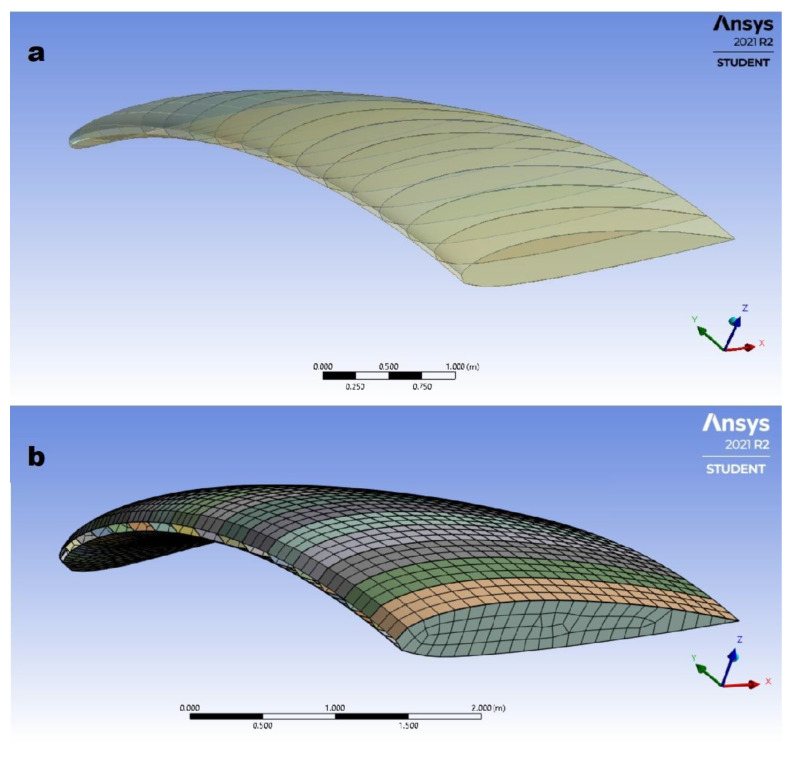
The model design: (**a**) geometry of a half of the considered paraglider; (**b**) FEM mesh.

First, a simplified geometry of the paraglider was created using the ANSYS Design Modeler program. In order to decrease the time of calculation and the number of elements creating a mesh, only half of a paraglider wing was generated. The final geometry (Figure 6a) contained lower and upper surfaces of the wing and ribs of an airfoil shape.

Before exporting to the meshing program, the surfaces creating lower and upper cover of the paraglider were split to create smaller faces connecting the closest ribs. This operation allowed better dimensioning of the mesh in the following steps, and therefore, resulted in a better quality of the created mesh (Figure 6b).

When the geometry was created it was possible to generate the mesh. The following methods were used.

To determine the geometrical model, the wing contour and airfoil cross-section were measured on the real structure. The dimensionless coordinates creating the airfoil profile are introduced in the elliptic equation to determine the coordinates of a particular point in the 3D Cartesian system. The rib profiles are created using the smoothing curves of the spline type.

Edge sizing is a method of dimensioning the mesh based on the precise determination of the number of nodes on selected edges. In the case under consideration, each of the edges defining the upper and lower profile contours were divided into 24 parts.

Face sizing is a method of dimensioning the mesh, consisting of determining the size of the cells on the indicated surfaces. The method was used to create the shape and dimensions of the ribs. No specific cell edge lengths were specified.

The shell element type mesh consisting of 3933 elements was generated (Figure 6b). It was characterized mainly by structural elements. However, due to geometry characteristics, some of elements remained unstructured.

Due to limited access to the full version of ANSYS and in order to shorten the time of calculation, a coarse mesh was decided to be applied. However, according to the mesh metrics (element quality = 0.89; skewness = 0.11; and aspect ratio = 1.14), the quality of the mesh secures the results. Moreover, additional calculation was performed for one case with dense mesh, and compared to the results, further described in this research. It was found that the results are comparable.

### 5.2. Numerical Simulation

Let us next introduce the material properties and environmental conditions characterizing the case under analysis.

As a paraglider is all a sewn object, all the elements are connected to each other. Therefore, it was important to model the common edges as the contact regions.

**Figure 7:**
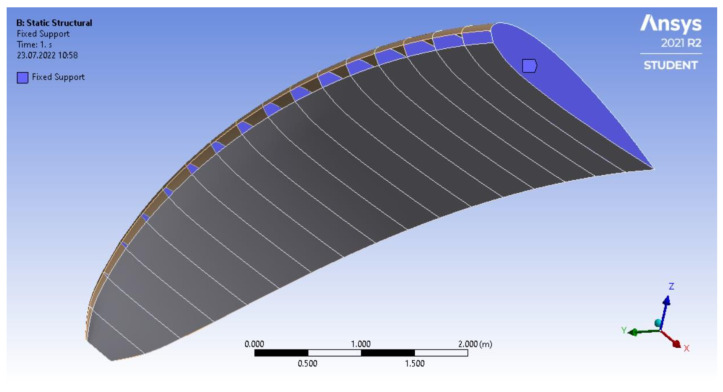
Attachment of the support for the numerical calculation.

To reduce the number of degrees of freedom and approximate the real conditions, a fixed support was applied on the faces that responded to the shapes of the ribs in the analyzed paraglider, Figure 7. The simplification determines the real conditions because the ribs are practically immovable during the flight.

As mentioned in Section 3.2, the mechanical and porosity properties obtained during the tests were a basis for the numerical simulations.

Let us consider the porosity properties to adjust the most proper results in the meaning on the pressure drop when testing compared to the actual conditions that could be met in air.

**Figure 8:**
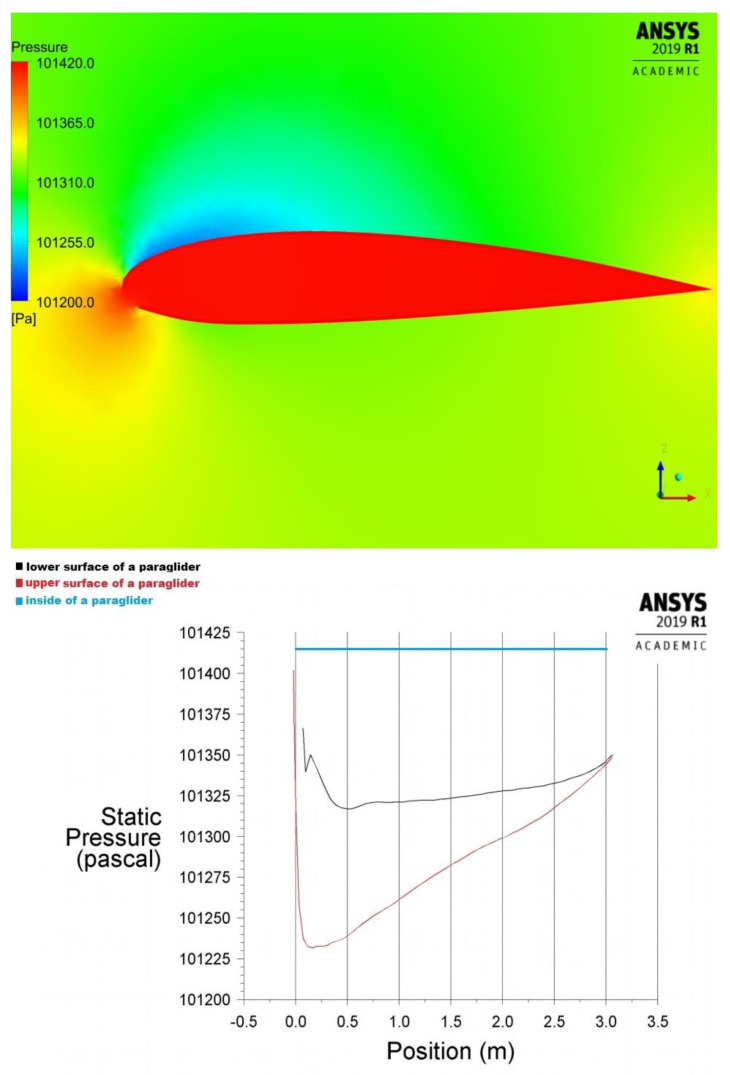
Pressure acting on a material during a flight of a paraglider (based on [9]).

Therefore, the pressure distribution over a paraglider covered with an impermeable material obtained by the ANSYS Fluent program during the above mentioned previous analysis [8,9] was considered, Figure 8.

The case [9] considered undisturbed flight conditions, where the velocity of a paraglider v = 45 km/h, the angle of attack α = 6°, the air temperature T = 300 K, and pressure p = 101,325 Pa. The air was considered as ideal gas.

The pressure distribution in Figure 8 is the most advantageous due to the paraglider’s principle of flight. A suitable lifting force is only created when the internal pressure is a constant of the maximal value. The lower pressure or the disturbed distribution inside the wing causes a rapid reduction in lift force and the unpredictable instability during the flight.

According to [4], the maximal pressure difference between air inside the wing and the upper surface was equal to 185 Pa.

However, the presented pressure distribution is applied to undisturbed flight conditions. Increased overload can be caused, for example, by a wind blowing or an operation of a pilot, and has a noticeable impact on pressure distribution and tensile forces acting inside the paraglider material. Thus, the pressure difference should be multiplied by a maximal overload during the flight.

Based on a plot created during an actual parachute wing-type drop recorded with an electromechanical flight data recorder, the average overload during the opening of a wing-type parachute is around 5.6 g (the name of a parachute manufacturer and a model of the parachute were not allowed to be published due to a business secret). Overloads during parachute drops are the highest that can be achieved during a usage of the analyzed type of a flying objects. In normal flight conditions, a paraglider is not subjected to such high overloads. However, for safety, it was decided to assume higher overload values than can be achieved.

According to the above conclusions, the analyzed materials were subjected to the maximal pressure drop during the flight equal to 1036 Pa. Therefore, the test results of air permeability for a pressure drop equal to 1500 Pa were chosen to be the most proper for the further analysis.

**Figure 9:**
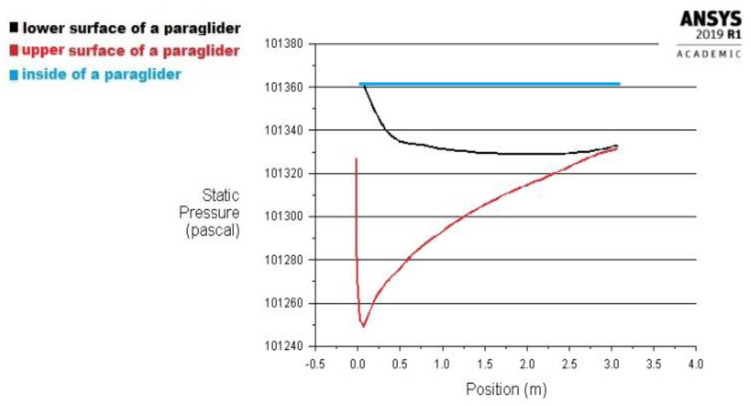
Pressure change vs. the distance from the leading edge in the symmetry plane (paraglider made of air-permeable material, referring to sample no. 9).

In the paper were considered the pressure distributions over a paraglider made of the impermeable material (based on Figure 8 referring to samples: 1–8 and 10), and material of air permeability equal to 1.74 m/s under 1500 Pa (based on Figure 9, referring to the sample 9).

Maximal pressure difference changes on the upper and lower surfaces of the considered materials in the symmetry plane are compiled in the Table 5. The results were achieved during a CFD analysis based on the previous research [8,9].

The above listed distances from the leading edge and the corresponding values of pressure are referring to the symmetry plane. However, the chord size decreases with distances from the symmetry plane. The pressure distribution on the upper surface of the wing was calculated in respect to the actual geometry of the analyzed paraglider and introduced to the program for the calculation.

Finally, the anisotropic characteristics of the woven materials that were achieved and analyzed in the course of physical testing (cf. Section 4.1 and Section 4.2) were assigned to the case under study in the ANSYS Structural program.

## 6. Simulation Results

The numerical calculations allow us to determine the results regarding deformation, stress, and strain of the analyzed materials (Table 6).

According to the Table 6, the lowest deformation and strain values were achieved for the material number 9. The considered sample is one of the strongest among the compiled results (only the sample number 10 achieved higher breaking force result, cf. Section 4.2). However, the main reason for the obtained values is that the material was subjected to the lowest pressure difference, which is caused by an increased air-permeability value.

When a bigger pressure acting on a material was considered (samples 4, 6, and 10), the deformation and strain decreased with increasing of the tensile strength of a material.

However, the stress value was determined by the following main factors:

- The thinner the material, the bigger the stresses that were accumulated. Sample no. 4 was characterized by the highest stress values among all the materials. Material no. 9 was of the same thickness; however, it was subjected to lower pressure acting on its surface.
- The stronger the material, the bigger the stresses that were accumulated. Sample number 10 was subjected to the stress of 181 kPa bigger than the sample number 4 (when the average values are considered); this is consistent with the strength of materials—the stiffer materials transfer more stresses than the elastic ones when the support is rigid.

**Figure 10:**
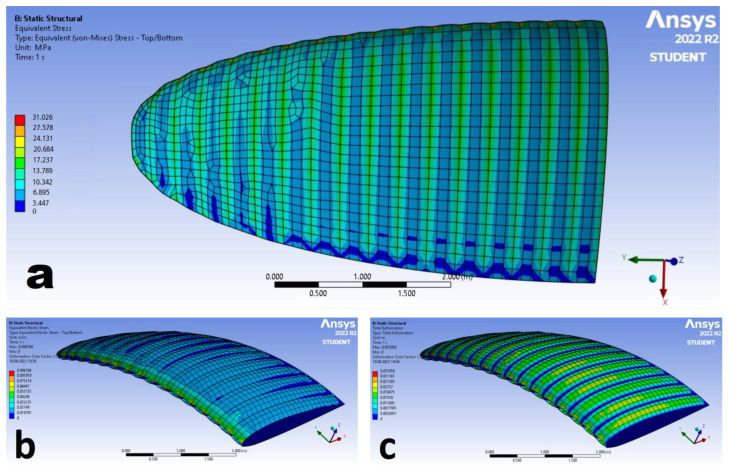
The exemplary distributions of (**a**) stress; (**b**) strain; and (**c**) deformation over the considered wing.

The results are similar for all the considered materials and differ mainly by the values, and the distributions were comparable. Therefore, the exemplary stress, strain, and deformation distributions are presented in the Figure 10.

According to Figure 10a, the biggest stresses were observed inside the material contacting the ribs. The biggest deformation and strain were observed in the regions between ribs, and the smallest in the contact areas with the fixed supports (Figure 10b,c). Their highest intensity was observed on the leading edge of the analyzed paraglider. This is probably caused by an absence of the attachment points on the considered edge.

The maximum values of stress, strain, and deformations over the paraglider were marked with the red color, which is almost not visible in Figure 10. Therefore, it can be concluded that the maximal values are not the most representative ones. The biggest repetitive values of stress, strain, and deformation could be assumed to be the representative maximal values, which do not coincide with the maximal parameters. Their averaged values, based on the visual results, are compiled in Table 7.

Based on the averaged maximal representative values of stress, strain, and deformation listed in the Table 7, it can be concluded that, in the sense of proportion, they were comparable to those compiled in Table 6. However, the values were much smaller than the absolute maximums.

The stress values decreased around 35% (when impermeable materials considered) and 25% (sample no. 9) compared to the maximums listed in Table 6. Whereas the strain decreased around 30–35% for all the samples, and the deformation was around 20% smaller.

When compared to the Table 3, all the obtained–absolute maximal values and representative maximal values of stress, strain, and deformation were far from values that could damage a paraglider (the safety factor of stress was around 3 for the paraglider-type fabrics and 6 for the parachute-type fabrics).

## 7. Conclusions

The aim of this paper was to determine experimentally and numerically the strength characteristics concerning the lightweight paraglider wing with Fourier transform infrared spectroscopy of applied materials. The samples define the wide range of materials covering the wing, as well as the parachute fabrics, and are representative for these structures. The materials were tested using the spectroscopy technique to determine the FTIR spectra. The samples differ in the content of certain characteristic groups. The data obtained based on the FTIR spectroscopy analysis were not implemented directly to the ANSYS program. However, FTIR spectroscopy results, when compared to the tensile results, give a full overview of the material characteristics. Therefore, it had a significant impact on the consideration of this research.

The air permeability of almost all the analyzed samples was close to zero with the exception of only one material. The permeability of this sample is greater than in other cases, but it is still very low. To secure the repeatability of results, the samples were subjected to different pressure drops between 100 Pa and 1500 Pa. The obtained results are very advantageous because the impermeable fabrics secure the high and stable pressure inside the paraglider wing.

The only problem was determining the sufficient strength characteristics of the material applied. The representative and determinable material parameter is the Young’s modulus E. Summarizing the test results, the paraglider samples were characterized by the slightly decreased mechanical properties compared to the parachute samples.

Based on the FTIR Spectra and following laboratory tests, two fabrics coated with polyurethane (i.e., samples 4 and 6) and two fabrics coated with silicone (samples 9 and 10) were chosen to be considered in the further ANSYS Structural analysis.

The material characteristics determined during the tests are the input data for the subsequent theoretical analysis. The numerical model of paraglider wing is based on a 3D geometry from a previous research, but the type of calculation is different. Stress, strain, and deformation distributions over the 3D model were determined using the ANSYS Structural program and the finite elements method. The tensile forces in the covering material are created by the pressure difference between the air inside and surrounding the wing. The pressure distribution over a paraglider structure covered with impermeable and permeable materials was determined using the ANSYS Fluent program.

To determine the strength correctly, we introduced two basic parameters: the absolute maximal values and the representative values that are the biggest repetitive values of stress, strain, and deformation. Introducing the safety factors of stresses for the paraglider (around 3) and the parachute fabrics (around 6), and analyzing the distributions of strength parameters, we conclude that the values were far from the breaking level for the paraglider wing. Thus, the materials covering the wing secure the strength adequate for the expected requirements.

The method applied is an advanced composition of experimental investigations and numerical modeling; that is, the theoretical model is supplemented by the tests of material parameters. Its advantage is the multiparametric and precise determination of strength characteristics. It follows that the proposed procedure is a promising tool to determine and evaluate the strength characteristics of fabrics covering the paraglider wing.

The shape and material properties can be optimized using two alternative methods: the theoretical minimization of an objective functional or the multistage tests of different structural parameters. The first procedure consists in the formulation of the proper objective functional, which is difficult with respect to the complex analysis of the wing. We introduce the different state variables, that is, the pressure in flight dynamics and the stress/strain/displacement in problems concerning the strength of materials. The comprehensive testing of material properties connected with the numerical modeling seems to be the only applicable method.

## Figures and Tables

**Table 1:** Basic characteristics of the analyzed materials.

Sample	Mass (g/m^2^)	Thickness (mm)	Number of Threads/dm	
			**warp**	**weft**
1	34	0.05	560	580
2	42	0.07	460	500
3	32	0.05	420	460
4	26	0.05	420	580
5	38	0.09	420	480
6	38	0.09	420	460
7	29	0.04	420	460
8	26	0.04	420	480
9	36	0.05	520	520
10	42	0.08	510	500

**Table 2:** Characteristic functional groups registered by FTIR analysis of the analyzed samples [20,21,22,23,24,25].

Peak Assignments and Type of Vibration	Wave Number [cm^−1^]
ν O-H w s	3300–3330
*v_s_* (C–H), *v*(=C–H_vw_)	3100–3050
stretching in Si-CH3	2960
*v*_as_ ν CH3s s	2935
*v*_as_ ν CH2s s	2852
*v* (C=O)	1735–1690
*v*(C=C) in aromatic rings	1680–1550
ν NC=O m vs	1631
δ N–H w s	1537
*d* N–H, ν C–N	1515
δ CH2vw w	1455
sv (C=C) aromatic	1441
*m* NCO in phase/CH2	1334–1333
ν C–N–w	1276
m (O=)C–O–C stretch/Urethane C–O stretch	1250–1248
*v*_m_(–C–O) or *d*_m_(–CH_2_–), *v*_w, m, vw_(–C–H, –CH_3_);	1285/1244
CH3 deformation in Si-CH3	1260–1250
*v* C-O	1183–1153
*v* C-O	1153–1123
ν*a* C–N–C	1155–1145
ν C–OH vs. *v*(–C–H)	1058–1056
*v* (O–C–O, *νa* C–O–C), *v*(C–C), *v*_m,vw_(–C–O),	1091–1020
*v* Si-O-Si	1074–1005
*v* C=C	958
ν OC–C s s, *w* νs C–N–C	871–865
−CH3 rocking and Si-C stretching in Si-CH3	796–789
*v, sv* C–N	765–760
ν CH2	731–727
γ N–H–m	686
δ NC=O–w	576

Abbreviations: *v*—stretching vibrations; *d*—deformation vibrations; *s*—symmetric; *as*—asymmetric; *st*—strong; *w*—weak; *vw*—very weak; *m*—medium; *sv*—skeletal vibration; and *a*—axial.

**Table 3:** Fabric characteristics obtained during the laboratory tests.

Sample	Mass (g/m^2^)	Thickness (mm)	Max. Force during Elongation (daN)		Elongation at Break (%)		Air Permeability (mm/s)				
			Warp	Weft	Warp	Weft	100 Pa	125 Pa	1500 Pa	2000 Pa	2500 Pa
**1**	34	0.05	34	32	21	20	0.00	0.00	0.00	0.03	0.02
**2**	42	0.07	35	35	26	25	0.00	0.00	0.00	0.05	0.07
**3**	32	0.05	25	33	24	25	0.00	0.00	0.00	0.00	0.00
**4**	26	0.05	25	22	24	22	0.00	0.00	0.00	0.05	0.06
**5**	38	0.09	38	33	25	25	0.00	0.00	0.00	0.00	0.00
**6**	38	0.09	38	33	26	25	0.00	0.00	0.00	0.00	0.00
**7**	29	0.04	25	33	24	25	0.00	0.00	0.00	0.06	0.08
**8**	26	0.04	25	22	24	23	0.00	0.00	0.00	0.00	0.00
**9**	36	0.05	40	40	26	26	0.00	0.00	1.72	2.30	2.44
**10**	42	0.08	42	42	27	27	0.00	0.00	0.00	0.06	0.08

**Table 4:** The obtained Young’s modulus values.

Sample	E (MPa)	
	Warp	Weft
**4**	815	810
**6**	582	536
**9**	885	862
**10**	669	657

**Table 5:** Pressure difference changes on the upper and lower surfaces of the considered materials in the symmetry plane.

Samples: 1, 2, 3, 4, 5, 6, 7, 8, 10								
**Distance from the leading edge**	**0.125 m**	**0.500 m**	**1.000 m**	**1.500 m**	**2.000 m**	**2.500 m**	**2.750 m**	**3.000 m**
**Upper surface**	10,365 Pa	980 Pa	857 Pa	756 Pa	644 Pa	560 Pa	476 Pa	392 Pa
**Lower surface**	420 Pa	560 Pa	532 Pa	504 Pa	487 Pa	465 Pa	420 Pa	392 Pa
**Sample 9**								
**Distance from the leading edge**	**0.083 m**	**0.500 m**	**1.000 m**	**1.500 m**	**2.000 m**	**2.500 m**	**2.750 m**	**3.000 m**
**Upper surface**	644 Pa	588 Pa	476 Pa	364 Pa	336 Pa	280 Pa	224 Pa	196 Pa
**Distance from the leading edge**	**0.375 m**	**0.500 m**	**1.000 m**	**1.500 m**	**2.000 m**	**2.500 m**	**2.750 m**	**3.000 m**
**Lower surface**	140 Pa	168 Pa	196 Pa	196 Pa	196 Pa	196 Pa	185 Pa	168 Pa

**Table 6:** The minimum, average, and maximum values of stress, strain, and deformation of the considered cases.

Sample	Stress [MPa]			Strain [%]			Deformation [m]		
	Min.	Av.	Max.	Min.	Av.	Max.	Min.	Av.	Max.
**4**	0.000	4.138	31.026	0.0	1.0	9.7	0.000	0.009	0.035
**6**	0.000	2.455	18.37	0.0	0.9	8.4	0.000	0.008	0.032
**9**	0.000	2.492	21.84	0.0	0.6	7.0	0.000	0.006	0.028
**10**	0.000	2.636	19.39	0.0	0.8	7.7	0.000	0.007	0.030

**Table 7:** The biggest representative (repetitive) values of stress, strain, and deformation based on the visual results; averaged.

Sample	Stress [MPa]	Strain [%]	Deformation [m]
**4**	20.90	7.0	0.027
**6**	12.26	5.6	0.025
**9**	16.99	4.7	0.022
**10**	12.39	5.4	0.023

## Data Availability

Data sharing not applicable.
